# Psychometric Properties of the Questionnaire Epistemic Trust in People With Mild to Moderate Intellectual Disabilities or Borderline Intellectual Functioning

**DOI:** 10.1111/jar.70111

**Published:** 2025-08-21

**Authors:** Suzanne D. M. Derks, Annelies de Bildt, Veerle M. M. Andries, Saskia Knapen, Paula S. Sterkenburg

**Affiliations:** ^1^ Department of Clinical Child and Family Studies & Amsterdam Public Health Vrije Universiteit Amsterdam Amsterdam the Netherlands; ^2^ Department of Child and Adolescent Psychiatry, University Medical Center Groningen University of Groningen Groningen the Netherlands; ^3^ Accare Child Study Center Groningen the Netherlands; ^4^ The Research School of Behavioral and Cognitive Neurosciences University of Groningen Groningen the Netherlands; ^5^ Stichting Odion Wormer the Netherlands; ^6^ TOPP‐zorg Zeist the Netherlands; ^7^ Bartiméus Doorn the Netherlands

**Keywords:** epistemic trust, intellectual disability, psychometrics, self‐report

## Abstract

**Background:**

To assess epistemic trust in people with intellectual disabilities, we adapted the Questionnaire Epistemic Trust (QET) for people with mild to moderate intellectual disabilities or borderline intellectual functioning (MMID/BIF).

**Method:**

We investigated the factor structure, the reliability and construct validity in 147 adults.

**Results:**

We replicated the 4‐factor structure, after excluding four items with low factor loadings. Internal consistency was *α* = 0.58 for Hypervigilance, and ranged from *α* = 0.74 to 0.81 for the other subscales. Subscale test–retest reliability ranged from 0.504 to 0.747. No convergent validity was found with the Reflective Functioning Questionnaire (RFQ). Discriminant validity was confirmed with the Scale of Emotional Development‐Questionnaire (SED‐Q), Scale of Emotional Development‐Short (SED‐S) and Autism Spectrum Quotient‐10 (AQ‐10), but not with General Social Trust (GST).

**Discussion:**

The QET is promising for assessing epistemic trust of people with MMID/BIF at subscale level. Refining the items with a figurative expression seems needed.

**Summary**

- To provide effective support for people with intellectual disabilities, it is important to understand whether they perceive and interpret the people who provide such support as trustworthy.
- Instruments to assess this trust in the knowledge and expertise of others were not available for people with intellectual disabilities.
- The current study showed that the subscales of an adapted version of the Questionnaire Epistemic Trust (QET) is promising for assessing this trust in people with mild to moderate intellectual disabilities or borderline intellectual functioning; although more research is needed.

## 1 Introduction

Epistemic trust, introduced by Fonagy and Allison (2014), refers to the capacity to consider conveyed knowledge as trustworthy, relevant to the self and generalisable to other contexts. Epistemic trust can be perceived as a trait‐like predisposition of a person characterised by specific perceptions, thoughts, feelings and behaviours in specific situations (Knapen et al. 2022). This predisposition is reflected in how a person perceives and interprets other people as being trustworthy, in their basic cognitions on the competency and trustworthiness of others, in their basic trust, curiosity and trust in social relations and in how they behave regarding collaboration and openness to the information and expertise of other people. Epistemic trust develops in a context of secure attachment (Fonagy and Allison 2014), where learning from others occurs through finding a balance between epistemic trust and epistemic vigilance (i.e., an instinctive doubt toward others' information that may be potentially harmful, misleading, or inaccurate; Fonagy and Allison 2014; Knapen et al. 2022). When persons have experienced persistent aversive childhood experiences, this balance is disturbed (Fonagy et al. 2017, 2019). This means that a person either relies too much on others and is too open for their information (i.e., epistemic credulity), or that they tend to trust no information from others at all (i.e., epistemic mistrust). These tendencies arise from their (mis)interpretation of the intentions and expertise of others, or a combination of both, and may even coexist simultaneously (Campbell et al. 2021).

Through the development of epistemic trust within secure attachment bonds, a foundation is created for meaningful interactions in future relationships. This is especially relevant in relationships in care situations with professionals (e.g., professional caregivers, therapists), where epistemic trust is essential for persons to learn from knowledge that is provided within care‐related relationships (Fonagy and Allison 2014; Knapen et al. 2020, 2022). Although the majority of the earlier literature has focused on psychotherapeutic relations (Fonagy and Allison 2014), it has been argued that epistemic trust may play a role in any care situation in which the trust in others' perspectives and expertise is crucial (Knapen et al. 2020; Riedl et al. 2023). To provide adequate care in these situations, based on the persons' abilities and needs, understanding their level of epistemic trust would be helpful. Care professionals could then integrate addressing epistemic (mis)trust in their relationship with the person in a way that fits their needs in order to improve the extent to which the person can benefit from the care provided (Knapen et al. 2023).

In particular, in the care for people with intellectual disabilities, the role of epistemic trust may be of significant importance. For people with intellectual disabilities, care relationships are present both in everyday life (such as with professional caregivers) and in more specific care situations (such as in therapy), where they often have to rely on information from these professionals (Giesbers et al. 2019). At the same time, people with intellectual disabilities often have difficulties in adequately interpreting social cues and accurately judging social situations, leading to the risk of easily being misled by others (DSM‐5‐TR, American Psychiatric Association 2022). This indicates a limited development of adaptive epistemic trust. This difficulty in assessing whom to trust can make social relationships particularly challenging. Epistemic trust develops in close connection with other developmental domains, such as language, reasoning and social development. Since intellectual disabilities often cause delays or impairments in these areas (APA 2022), epistemic trust may also be affected. Additionally, people with intellectual disabilities are at greater risk of experiencing difficulties in parent–child relationships, which can lead to higher rates of insecure attachment over time (Hamadi and Fletcher 2021). Insecure attachment, in turn, is a known risk factor for developing both epistemic credulity and epistemic mistrust. Many factors have been reported that may lead to insecure attachment patterns in children with intellectual disabilities, among which a higher risk for institutionalisation (Green and Goldwyn 2002), parental difficulties in identifying and responding to the needs of the child (Giltaij et al. 2015; Vandesande et al. 2019), more parental mental health problems affecting the interaction (Aktan et al. 2020; Singer et al. 2006), parental feelings of grief and loss regarding their child affecting the sensitivity and responsivity of parents (Di Renzo et al. 2020; Feniger‐Schaal and Oppenheim 2013; Fletcher 2016; Oppenheim et al. 2024), or poor care and early abuse (Fletcher and Gallichan 2016). In addition, people with intellectual disabilities are more likely to experience negative or even traumatic encounters within (medical) healthcare settings (McCormick et al. 2021), which may further undermine the development of epistemic trust in professionals. Therefore, more research is needed to understand epistemic trust, epistemic credulity and epistemic mistrust in people with intellectual disabilities.

In order to assess epistemic trust in people with intellectual disabilities, instruments are needed. Recently, Knapen et al. (2023) designed the Questionnaire Epistemic Trust (QET) to assess the important clinical features of epistemic trust in a community and a clinical population. Aiming for a user‐friendly, brief questionnaire, Knapen et al. (2023) succeeded in developing a 24‐item questionnaire with four subscales of six items each: (1) Hypervigilance, (2) Curiosity/Openness, (3) Expectation of help and (4) Openness to help. The items were formulated based on the consensus definition of epistemic trust by experts in the field of attachment (Knapen et al. 2022) as described above. Their research into its psychometric values indicated the questionnaire to be promising.

Given the potential of the QET, the question was raised as to whether the QET could be used in people with mild to moderate intellectual disabilities or borderline intellectual functioning, to gain a preliminary understanding of their epistemic trust. Therefore, we adapted the wording of the QET to a level of inclusive language that was comprehendible for people with mild to moderate intellectual disabilities or borderline intellectual functioning. Second, we assessed the adapted QET to obtain insight into its psychometric properties in a sample of adults with mild to moderate intellectual disabilities or borderline intellectual functioning. In the current study, we investigated the factor structure, internal consistency and test–retest reliability. Additionally, we assessed convergent and discriminant validity based on associations of the adapted QET with measures of a related concept. Convergent validity was assessed in relation to mentalising, measured with the Dutch Reflective Functioning Questionnaire for adults with mild intellectual disabilities or borderline intellectual functioning (RFQ; Derks, Willemen, et al. 2023). Discriminant validity was assessed in relation to three different concepts, that is, general social trust as measured with the General Social Trust (GST; Åslund et al. 2010), emotional development as measured with both the Scale Emotional Development‐Short (SED‐S; Sappok et al. 2016) and the Scale of Emotional Development‐Questionnaire (SED‐Q, Vonk 2021), and autistic traits as measured with the Autism Spectrum Quotient (AQ‐10; Kent et al. 2018).

We expected to replicate the factor structure of the original QET and find similar levels of reliability compared to the original QET. Regarding convergent validity, we expected at least moderate correlations between the QET and the RFQ, since the underlying constructs are slightly different yet related in attachment theory. With the measures of general social trust and emotional development, we expected weak correlations as these are expected to measure conceptually separate constructs (discriminant validity). Regarding autistic traits, our expectations are less clear. Although autism is considered a separate construct, it has been associated with a tendency to misread others due to difficulties in interpreting social cues, nonverbal communication and emotional expressions (Embregts and Van Nieuwenhuijzen 2009). Because both autistic traits and low epistemic trust may manifest in similar social difficulties, it is important, from a differential diagnostic perspective (Sarr et al. 2025), to investigate whether these constructs are empirically distinct. We therefore included a measure of autistic traits to assess discriminant validity: correlations between measures of epistemic trust and autistic traits are expected to be weak, although they may approach values more indicative of convergent validity.

## 2 Methods

### 2.1 Participants

A total of 147 adults with mild to moderate intellectual disabilities or borderline intellectual functioning participated in the study. Inclusion criteria were age ≥ 18 years and a known indication of mild to moderate intellectual disability (IQ 35–70) or borderline intellectual functioning (IQ 70–85). People with an additional visual impairment (blind and partially sighted) were included. Seriously ill people (e.g., when hospitalised) or people who were not able to understand questions read to them in Dutch were excluded. Other exclusion criteria were not applicable. The sample characteristics are displayed in Table 1.

**TABLE 1:** Sociodemographic characteristics of the participants (*N* = 147).

Characteristic	*n*	%
Gender		
Female	86	59
Male	61	41
Country of birth		
The Netherlands	110	75
Belgium	23	16
Other	14	9
Vision^a^		
Visually impaired	34	25
Not visually impaired	103	75
Level of intellectual functioning		
Borderline (IQ 71–85)	21	15
Mild (LVB; 50–70)	98	71
Moderate (MVB; IQ 35–49)	15	11
Unknown	5	3
Autism		
Classification	28	20
No classification	110	80
Support per week		
0–2 days	48	33
3–6 days	18	12
7 days	80	55
Support per day		
< 12 h	93	63
≥ 12 h	54	37

*Note:* Missing data: gender: 1; country of birth: 1; vision: 11; exact level of intellectual functioning: 9; autism: 10; support per week: 2; support per day: 1. For participants without reported level of intellectual disability, inclusion was based on information at referral indicating mild to moderate intellectual disabilities or borderline intellectual functioning.

^a^ For one person, it was unknown whether they had a visual impairment.

Post hoc power analysis showed that for the factor analysis with 24 items and 4 factors, the total sample size of 147 yielded sufficient power (*β* = 0.80; *α* = 0.05) to reliably detect at least a CFI of 0.90 (with average factor loadings of 0.62 and average factor correlations of 0.55) and a RMSEA of 0.05 (Arifin 2024). The sample size for test–retest reliability (*n* = 45) was sufficient to detect a minimum acceptable reliability (ICC) of 0.50, with an expected reliability of 0.75, an alpha of 0.05, a power of 0.80 and two repetitions per subject (*k* = 2; Arifin 2024).

### 2.2 Procedure

The participants for this study were recruited between December 2022 and December 2023, in care organisations for people with intellectual disabilities in the Netherlands and Flanders, Belgium. Potential participants and their professional caregivers or family members received a letter from the organisation with an information brochure, accompanied by an information video, developed in co‐creation by researchers and co‐researchers (adults with mild intellectual disabilities or borderline intellectual functioning). The information brochure was based on the ‘easy‐to‐read’ standards (Inclusion Europe 2007) and the ‘easy writing’ guideline from the ‘Language for all’ method (Reichrath and Moonen 2022). Additionally, it contained visual aids to enable people with mild to moderate intellectual disabilities or borderline intellectual functioning to understand the study and the study procedure. For visually impaired people, the information brochure was converted into an audio recording. In addition, the research was made known within client councils, newsletters, via the broad networks, on websites (e.g., www.klik.org) and via social media. Participants could register for the study via their care organisation or could contact the researchers themselves.

Participants who registered were supported by an independent researcher, that is, research‐assistant, student(‐assistant), or (educational) psychologists (in Dutch: Orthopedagogen) during data‐collection. They were asked to read and sign a consent form before the start of the study. The consent form was read out if needed. To help participants in their decision to participate and to provide consent, parents, professional caregivers or other representatives were informed so that they could assist the eligible persons in their decision. In cases of legal incapacitation, the legal representative provided consent. The professional caregiver was also asked to sign a consent form as they filled in the SED‐S (see Measures for further information).

Data were collected by using Qualtrics software. The questionnaires were completed via a link on a tablet or laptop/computer. When clients required assistance in completing the questionnaires, support was provided by the independent researcher following a standardised protocol. Besides assisting the participants, these independent researchers were trained in assessing the SED‐Q with participants and SED‐S with professional caregivers. The first assessment took approximately 90–120 min for each participant and 60–90 min for the professional caregiver. A randomly selected group of participants (*n* = 60) and their professional caregivers were asked to fill in the QET, GST and SED‐Q three weeks after the first appointment to assess test–retest reliability. This second assessment took approximately 90 min per participant and 60–90 min for the professional caregiver. Eventually, 147 participants and 136 professional caregivers participated, of which 45 participants participated in a second assessment appointment. Participants received a small gift for their participation at the end of the first appointment, and in case of test–retest participants at the end of the second appointment as well.

The Medical Ethics Committee of the University Medical Centre Amsterdam location VUmc, the Netherlands determined that our study did not fall under the scope of the Medical Research Involving Human Subjects Act (WMO; 2022.0466). Ethical approval was provided by the Institutional Review Board of the Faculty of Behavioural and Movement Sciences of the Vrije Universiteit Amsterdam (VCWE‐2022‐140). The study was pre‐registered (Derks, Andries, et al. 2023).

### 2.3 Measures/Instruments

#### 2.3.1 Epistemic Trust

The Questionnaire Epistemic Trust (QET) is a self‐report questionnaire on the degree of epistemic trust (Knapen et al. 2023). The QET, already available in Dutch and English, consists of 24 items that can be divided into four subscales: Hypervigilance, Curiosity/Openness, Expectation of help and Openness for help. The questionnaire is adapted according to the guidelines of Beaton et al. (2000). The steps were: (1) one project team member (S.D.M.D.) and two scientist practitioners familiar with working with people with intellectual disabilities independently performed adaptations and compared the adaptations till consensus was reached, (2) the adaptations were reviewed by co‐researchers representing the target group, that is, people with intellectual disabilities, (3) the adaptations were discussed (S.D.M.D.; P.S.S.) with the developer of the original QET (S.K.), and (4) the adapted QET was pretested with newly recruited people, that is, two people of all three cognitive levels (mild, moderate and borderline), one with and one without a visual impairment. Feedback was provided through an interview. This resulted in a final version that was again checked (V.M.M.A.; P.S.S.) with the author of the QET (S.K.). Statements are, for example: ‘I am open to information other people give me’ and ‘I don't just follow advice or tips from my professional caregiver about what I should do’. This final version was the starting point for the current study and is available upon request.

Similar to the original version, items of the adapted QET are scored on a 5‐point Likert scale ranging from 1 (‘completely disagree’) to 5 (‘completely agree’; Knapen et al. 2023). In accordance with recommendations in Kooijmans et al. (2022) on the amount of answer options given simultaneously in a Likert scale, answering was split into two steps. Participants could first choose to score ‘disagree’, ‘neutral’ or ‘agree’. The participants who chose ‘disagree’ or ‘agree’ were subsequently asked to specify the option they had chosen (i.e., for ‘disagree’ into ‘completely disagree’ or ‘disagree’ and for ‘agree’ into ‘completely agree’ or ‘agree’). After reverse scoring of negatively formulated statements, a higher score indicates a higher level of epistemic trust.

In a community sample, excellent validity and reliability have been reported for the original QET with acceptable model fit and good to excellent Cronbach's alphas ranging from 0.80 to 0.90 for the four subscales, and *α* = 0.91 for the total scale (*N* = 107, 98 female, *M*_age_ = 45.39; Knapen et al. 2023).

#### 2.3.2 General Social Trust

The Dutch version of the General Social Trust (GST; Åslund et al. 2010) was used to assess how participants think about people in general. Although the underlying concepts of the GST and the QET may sound related, the GST differs from the QET in its scope and in the object of trust. The GST aims to measure a broader concept, including trust in people, their intentions and social interactions and a world that is organised fairly, whereas the focus of epistemic trust is on acquiring knowledge or information accurately. The GST is a self‐report questionnaire that consists of six statements, to be scored on a 4‐point Likert scale, with answers ranging from ‘strongly agree’ (1) to ‘strongly disagree’ (4). Example statements are: ‘Most people can be trusted’ and ‘Most people try to be helpful’. For a total score (range 6–24), all items were summed. Items 2, 4 and 5 were formulated in a negative form and therefore were reversed for summation of the total score. A higher score indicated a higher level of general social trust. Fair internal consistency of the GST was found in the current sample (*α* = 0.68), which is consistent with what Åslund et al. (2010) have previously reported.

#### 2.3.3 Reflective Functioning

The Reflective Functioning Questionnaire (RFQ; Fonagy et al. 2016) was developed to measure inabilities of mentalising. The Dutch version of this self‐report questionnaire was adapted for people with intellectual disabilities in the Netherlands and then validated in this group by Derks, Willemen, et al. (2023). This Dutch adapted RFQ has 10 items (7 from the original RFQ, 3 additional ones), such as ‘I don't always know why I do something’ and ‘It is easy for me to know what other people are thinking and feeling’. Items are scored on a 7‐point Likert scale ranging from ‘strongly disagree’ (1) to ‘strongly agree’ (7). Factor analysis of this adapted version replicated the two factor solution of the original, resulting in the creation of two subscales: the subscale Self with items focused on the self and one's own feelings and thoughts (items 2, 3, 4, 5, 6 and 7) and the subscale Other with items focused on the self in relation to the feelings and thoughts of others (items 1, 8, 9 and 10). After reverse scoring of item 8, higher scores imply more uncertainty in reflective functioning. In accordance with the findings of Derks, Willemen, et al. (2023), the current study showed moderate Cronbach's alpha for the Self subscale (*α* = 0.75) and unsatisfactory Cronbach's alpha for the Other subscale (*α* = 0.59).

#### 2.3.4 Autism

The Dutch version of the Autism Spectrum Quotient‐10 (AQ‐10; Allison et al. 2012) screening tool was used to measure autistic traits. We applied the 10 items of the original self‐report questionnaire (Allison et al. 2012) on a 2‐point scale in accordance with Kent et al. (2018), who adapted the AQ‐10 for people with intellectual disabilities. Answer options were: ‘Yes’, ‘No’ or ‘Don't know’. Statements are, for example: ‘I find it easy to do more than one thing at once’ and ‘I find it difficult to work out people's intentions’. In the current study, a Cronbach's alpha of 0.31 was found for the total AQ‐10. After deleting poor functioning items (items 1, 2 and 8), Cronbach's alpha improved (*α* = 0.61) to a nearly fair level. Therefore, the mean score for autistic traits was based on the seven remaining items, with higher scores indicating more autistic traits. ‘Don't know’ answers were treated as system missing.

#### 2.3.5 Emotional Development

To assess emotional development, two scales were applied. First, the Scale of Emotional Development‐Questionnaire (SED‐Q; Vonk 2021; in Dutch: VEO) was administered with the participants themselves. Second, the Scale of Emotional Development‐Short (SED‐S, Sappok et al. 2016) was administered with a professional caregiver. As the professional caregiver version SED‐S was the basis for the self‐report version SED‐Q, we describe the instruments in that order here.

The Scale of Emotional Development‐Short (SED‐S; Sappok et al. 2016) is a semi‐structured interview with a (professional) caregiver or family member for assessing levels of emotional development in people with intellectual disabilities. It consists of 200 binary items about observable behaviours exhibited by the person within 2–12 weeks before the interview (Sappok et al. 2019). The Dutch version was used (Morisse et al. 2017). The items are grouped into eight domains: Relating to one's Own Body, Relating to Significant Others, Dealing with Change‐Object Permanence, Differentiating Emotions, Relating to Peers, Engaging with the Material World, Communicating with Others and Regulating Affect. Each domain covers the first five stages of emotional development (Adaption, Socialisation, Individuation, Identification, Reality and Awareness) and contains five statements per stage (total of 25 statements per domain; Sappok et al. 2016). Within every domain, the stage with the highest number of positively answered items is assumed to provide the best estimation of the level of emotional development. If two levels are rated with an equal number of items, the lowest is chosen as the domain score (Sappok et al. 2016), except if this level of development is implausible. To estimate the overall level of emotional development, domain scores are ranked from lowest to highest, with the fourth lowest score being the final score. A higher score indicates a higher overall level of emotional development. The SED‐S has been validated for people with mild to moderate intellectual disabilities or borderline intellectual functioning, showing a strong model fit, excellent internal consistency (*α* = 0.93) and construct validity across all subgroups (Flachsmeyer et al. 2023). In the current study, an excellent Cronbach's alpha of 0.91 was found.

The Scale of Emotional Development‐Questionnaire (SED‐Q; Mesker et al. 2025; Vonk 2021; in Dutch: VEO) was used to assess levels of emotional development among the participants. In order to empower people with intellectual disabilities to talk about their emotional development rather than others talking about them, the Dutch version of the SED‐S has been further developed into the Dutch SED‐Q that can be completed together with people with intellectual disabilities instead of with professional caregivers or relatives. This resulted in a questionnaire administered through a semi‐structured interview by a trained assessor (Vonk 2021). The SED‐Q contains the same eight domains as the SED‐S. Additional to the first five stages of emotional development in each domain in the SED‐S, two later stages of emotional development have been added to each domain in the SED‐Q: Social Individuation and Social Responsibility. As with the SED‐S, each stage within each domain consists of five statements; thus, each SED‐Q domain entails 35 statements (Vonk 2021). To reach domain scores and an overall score, the same procedure is applied as for the SED‐S. A higher score indicates a higher level of emotional development. In the current study, the SED‐Q showed excellent internal consistency (*α* = 0.94).

#### 2.3.6 Additional Information

Sociodemographic data were partially collected directly from the participants (e.g., gender, age, living and work situation), and partly via the professional caregiver or family member (e.g., level of intellectual, autism and visual impairment).

### 2.4 Data‐Analysis

We performed confirmatory factor analyses (CFA) in JASP 0.19.1.0 in order to test the factor structure of the QET (Knapen et al. 2023). We investigated both a 1‐factor model and a 4‐factor model, based on Knapen et al. (2023). The adequacy and acceptability of the model were determined based on the normed chi‐square (< 2 indicating good, 2 to < 3 indicating acceptable model fit), the root mean square error of approximation (RMSEA; < 0.05 considered good, ≥ 0.05 to ≤ 0.08 considered acceptable), the comparative fit index (CFI; values ≥ 0.9 to 0.95 considered acceptable, values > 0.95 considered good) and the standardised root mean square residual (SRMR; values < 0.08 considered good; Hu and Bentler 1999; Schweizer 2010). If called for, we improved the model based on the factor loadings of individual items, using the cutoff value of 0.30.

Next, the reliability of the 24‐item QET was determined. First, we computed internal consistency (Cronbach's alpha) following the guidelines of Ponterotto and Ruckdeschel (2007). Alphas could be unsatisfactory, fair, moderate, good or excellent, depending on the number of items per subscale (≤ 6, 7–11, ≥ 12) and the sample size of the study (*N* < 100, 100–300, > 300). Second, test–retest reliability of the 24‐item QET was assessed by calculating the Intraclass Correlation Coefficients (ICCs) (single rating [*k* = 2], absolute agreement, 2‐way mixed‐effects model, 95% confidence interval [CI]) of the total model and subscales. Values can indicate poor (< 0.50), moderate (≥ 0.50 to < 0.75), good (≥ 0.75 to < 0.90) and excellent (≥ 0.90) reliability. If the factor analyses led to an adjusted model, the above‐mentioned calculations for reliability were also conducted for the adjusted model. The factor analyses and internal consistency analyses were performed on cases with complete QET data. The test–retest reliability analyses were performed on cases with complete QET data at test and retest.

To investigate the construct validity, we determined the relation between the QET and the RFQ (convergent validity), and between the QET and the GST, the SED‐Q, the SED‐S and the AQ‐10 (discriminant validity). The associations between the total scores on the 24‐item QET and on these instruments were analysed by Pearson's correlation tests for continuous variables. Where correlations of approximately 0.50 or higher indicate convergent validity, correlations of 0.30 or lower indicate discriminant validity (Repke et al. 2024). Correlations between 0.30 and 0.50 can suggest conceptual overlap between the constructs. Again, if the model had been adjusted based on the factor analyses, convergent and discriminant validity were examined for the adjusted model. In case of missing data, these were handled using pairwise deletion.

## 3 Results

### 3.1 Factor Structure

As presented in Table 2, the results of the CFA indicated a poor model fit for the 1‐factor model (*χ* ^2^/df = 6.12, RMSEA = 0.187 [90% CI = 0.187–0.196], CFI = 0.808 and SRMR = 0.154). Deleting items with very low factor loadings (< 0.2; i.e., items 1, 3, 4 and 5 of the subscale Hypervigilance and item 20 of Openness for help) resulted in a fit that still was not acceptable for the 1‐factor model based on *χ* ^2^/df = 4.65, the RMSEA = 0.158 [90% CI = 0.146, 0.170] and SRMR = 0.132. Of all indices, only the CFI showed a good fit (0.907). Also, factor loadings of items 8 and 9 remained lower than the cutoff value of 0.30. Deletion of these items did not result in a better fit (*χ* ^2^/df = 5.18, RMSEA = 0.169 [90% CI = 0.156, 0.182], CFI = 0.914, SRMR = 0.132).

**TABLE 2:** Factor loadings and fit indices for the 1‐factor and 4‐factor QET (*N* = 147).

Factors	Item	1‐factor model	4‐factor model
Hypervigilance	QET_1	0.163	**0.376**
	QET_2	**0.328**	**0.723**
	QET_3	0.155	0.285
	QET_4	0.175	0.240
	QET_5	0.083	0.179
	QET_6	**0.415**	**0.867**
Curiosity/openness	QET_7	**0.507**	**0.595**
	QET_8	0.271	**0.373**
	QET_9	0.251	**0.332**
	QET_21	**0.654**	**0.588**
	QET_22	**0.722**	**0.940**
	QET_23	**0.659**	**0.689**
Expectation of help	QET_10	**0.696**	**0.903**
	QET_12	**0.798**	**0.899**
	QET_14	**0.701**	**0.658**
	QET_15	**0.477**	**0.432**
	QET_16	**0.607**	**0.748**
	QET_17	**0.745**	**0.606**
Openness for help	QET_11	**0.394**	**0.611**
	QET_13	**0.782**	**0.932**
	QET_18	**0.431**	**0.864**
	QET_19	**0.728**	**0.853**
	QET_20	0.079	0.265
	QET_24	**0.636**	**0.876**
Fit indices original model	Normed Chi‐square (*χ* ^2^/df)	6.12	1.67
	RMSEA	0.187	0.068
	CFI	0.808	0.909
	SRMR	0.154	0.107
Fit indices adjusted model	Normed Chi‐square (*χ* ^2^/df)	5.18	1.08
	RMSEA	0.169	0.024
	CFI	0.914	0.991
	SRMR	0.132	0.087

*Note:* The original models contain all items presented. The adjusted models only contain the items of which the factor loadings are printed in bold.

Furthermore, we investigated the model fit of the 4‐factor model. The results of the CFA instantly indicated a good model fit for the 4‐factor model with all 24 items (*χ* ^2^/df = 1.67, RMSEA = 0.068 [90% CI = 0.056; 0.079], CFI = 0.909, SRMR = 0.107). Most factor loadings were higher than 0.3; yet, four items had factor loadings below 0.3 (items 3, 4, 5 and 20). Deleting these items resulted in an excellent model fit (*χ* ^2^/df = 1.08, RMSEA = 0.024 [0.000; 0.045], CFI = 0.991, SRMR = 0.087) for the 4‐factor model with the 20 remaining items. Table 2 provides the factor loadings for the (adjusted) 1‐factor model and 4‐factor model.

### 3.2 Reliability

Internal consistency (*N* = 147) was unsatisfactory for subscale Hypervigilance (*α* = 0.58), and (almost) good to excellent for subscales Curiosity/Openness (*α* = 0.74), Expectation of help (*α* = 0.81) and Openness for help (*α* = 0.77) in the 4‐factor model. Internal consistency within the adjusted 4‐factor model varied from unsatisfactory to good, with slightly improved Cronbach's alphas for subscale Hypervigilance (*α* = 0.61) and subscale Openness for help (*α* = 0.78).

Test–retest (*n* = 45) of the subscales was poor to moderate/good, with an ICC of 0.461 (95% CI = 0.196–0.663) for Hypervigilance, 0.744 (95% CI = 0.580–0.851) for Curiosity/openness, 0.747 (95% CI = 0.583–0.853) for Expectation of help and an ICC of 0.729 (95% CI = 0.556–0.842) for Openness for help. For the adjusted 4‐factor model, ICCs became borderline moderate for Hypervigilance (ICC of 0.504; 95% CI = 0.254–0.691) and remained moderate for Openness for help (ICC of 0.688; 95% CI = 0.496–0.816). The number of items in the other subscales did not change.

### 3.3 Construct Validity

Table 3 shows the mean scores, standard deviations and correlations for the subscales of the QET and the other measures. No strong correlations between the QET and the RFQ were found, indicating no convergent validity. However, a nearly moderate correlation was found between the adapted Hypervigilance subscale of the QET and the RFQ Self. The correlations examined to investigate discriminant validity were mixed: the QET had low correlations with the SED‐Q, the SED‐S and the AQ‐10, but showed negative, moderate correlations with the GST.

**TABLE 3:** Means, standard deviations and correlations among QET subscales and the other measures (*N* = 147).

Measure		QET	QET	QET	QET
		Hypervigilance	Curiosity/openness	Expectation of help	Openness for help
	Mean (SD)	2.52 (0.66) 2.95 (0.91)	4.09 (0.72)	3.91 (0.80)	3.11 (0.87) 3.29 (0.94)
RFQ_Self	4.38 (1.36)	−0.126 −0.298 **	0.002	0.117	−0.137 −0.135
RFQ_Other	3.82 (1.33)	0.014 −0.111	−0.142	−0.005	−0.148 −0.159
GST	2.44 (0.59)	−0.420** −0.410 **	−0.284**	−0.386**	−0.195* −0.244 **
SED‐Q	4.34 (1.07)	0.072 0.232 **	0.212*	0.112	0.161 0.173 *
SED‐S	3.66 (0.85)	0.077 0.159	0.188*	0.094	0.091 0.090
AQ‐10	0.50 (0.22)	0.080 0.042	0.030	0.125	−0.012 −0.016

*Note:* Values and correlations in grey are from the adjusted 20‐item QET, affecting the subscales Hypervigilance and Openness for help. Correlations with the SED‐S and SED‐Q were based on a smaller sample size due to missing data on these measures (SED‐S: *N* = 135; SED‐Q: *N* = 133).

* *p* < 0.05.

** *p* < 0.001.

## 4 Discussion

This study investigated the psychometric properties of an adapted version of the QET (Knapen et al. 2023) for people with mild to moderate intellectual disabilities or borderline intellectual functioning. Adaptation with the involvement of all stakeholders resulted in a version with similar, yet adjusted 24 items of the original QET, all rephrased while still aligning with the original items and in language that was understandable for people with mild to moderate intellectual disabilities or borderline intellectual functioning.

Contrary to our expectations, the underlying structure of the original QET was not fully replicated. In our sample of 147 people with mild to moderate intellectual disabilities or borderline intellectual functioning, we did not find a well‐fitting 1‐factor solution. Deleting five items with low factor loadings (< 0.3) did not improve the fit. We replicated the 4‐factor structure, finding acceptable fit and even good fit when the four items with low factor loadings were deleted. However, the latter resulted in the presence of only three items in the subscale Hypervigilance. This subscale had poor internal consistency (*α* = 0.61), also with the original number of items (*α* = 0.58). These values were lower than for the original QET (*α* = 0.88 in the clinical population; Knapen et al. 2023). Additionally, test–retest reliability was poor for the Hypervigilance subscale (ICC 0.461) and reached borderline moderate reliability in the adapted three‐item version (ICC 0.504). The subscales Curiosity/openness, Expectation of help and Openness for help had acceptable to good internal consistencies (*α* ranging from 0.74 to 0.81), approaching the levels reported by Knapen et al. (2023; α ranging from 0.80 to 0.90 in the clinical population). Test–retest reliability of these subscales was moderate to good (ICC ranging from 0.688 to 0.747). These outcomes indicate that in its current form, the adapted 4‐factor QET shows promise as a tool for assessing epistemic trust in people with mild to moderate intellectual disabilities or borderline intellectual functioning, but further refinement is needed.

These refinements can be guided by the items that had low factor loadings and therefore affected the value of the 24‐item QET for people with mild to moderate intellectual disabilities or borderline intellectual functioning. These items were: *I quickly doubt the information others give me. I am not sure if it can be trusted* (item 1 Hypervigilance), *I am careful and pay close attention when someone tries to teach me something* (item 3 Hypervigilance), *I am careful of others giving me misinformation* (item 4 Hypervigilance), *I am careful and pay close attention when other people give me information* (item 5 Hypervigilance), *I am careful and pay close attention when my caregiver tries to teach me something (item 20 Openness for help)*. One explanation could be that not all items seemed to be optimally rephrased, resulting in low factor loadings. These low factor loadings may be explained by difficulties in translating certain words from the original QET, such as ‘misleading’, which resulted in more detailed but probably not accurate explanations of these words. Moreover, it was complicated to adapt figurative language (e.g., ‘be on my guard’) in a way that avoided literal interpretation by the target audience. In addition, some translations such as ‘pay close attention’ may not have achieved the desired associations among the participants as it can have had too positive connotations for a phrase that should rather evoke a certain level of vigilance. Finally, splitting an original QET item into two parts (e.g., adaptation of Item 1: *I am quick to doubt information that others give me. I am not sure if it can be trusted*) may not have led to the intended improvement in clarity or understanding. A renewed detailed examination of the adjusted items, knowing that they did not fit in the factor structure as intended, will be needed to give lead to further improving the adapted QET.

Another explanation could be that the Hypervigilance subscale may have a different meaning in people with moderate or mild intellectual disabilities or borderline intellectual functioning compared to a general population or a clinical population without intellectual disabilities. Some of the adaptive behaviour limitations associated with intellectual disabilities in the social domain (DSM‐5‐TR, American Psychiatric Association 2022) refer to gullibility and naivete. When people have experienced negative consequences of a gullible or naïve perspective on others, they may become more vigilant, perhaps resulting in a hypervigilant attitude after several negative experiences. Additionally, when people are being warned by others to be careful in trusting others, hypervigilance may occur. Both these sequences are well imaginable for people with moderate or mild intellectual disabilities or borderline intellectual functioning. Further research is needed to investigate the measurement invariance of the subscales across populations.

We expected the construct validity to be stronger than we found. We found low to moderate correlations of the 4‐factor QET with the GSTs, the SED‐Q and SED‐S as expected, which supports the discriminant validity of the QET as an instrument for a different concept than general trust in people or emotional development. Additionally, low correlations were found with autistic traits as measured with the AQ‐10, indicating that these concepts are not highly related. However, we expected at least moderate correlations between the QET and mentalising as measured with the RFQ; yet, we only found a significant but low correlation between the adapted Hypervigilance subscale and the RFQ Self. The negative low correlation between the adjusted Hypervigilance subscale and the RFQ Self subscale implies that the less hypervigilant one is on advices or intentions of others, the less uncertain one is about their own mental states. Both aspects share a general degree of certainty or doubt in dealing with knowledge, beliefs and intentions. The overall absence of an association between epistemic trust and mentalising, on the contrary, was surprising. Even though the constructs do not fully overlap, they seem related in attachment theory (Fonagy et al. 2019), and earlier research found higher correlations between the QET and the RFQ (Knapen et al. 2025). An explanation might be that epistemic trust is formed by social experiences (Cracknell et al. 2024) and may not be directly affected by the disability. In addition, the QET items can be answered by recalling concrete social situations that are applicable to the specific item. Reflective functioning, on the other hand, relies on more complex cognitive abilities such as meta‐reflection (Fonagy et al. 2016), and may therefore be more challenging for people with intellectual disabilities (Derks, Willemen, et al. 2023). This also applies to completing the RFQ, which requires a certain level of reflective capacity. This might explain that correlations for our specific target group are absent. In future studies, the association between epistemic trust, based on the improved QET and mentalisation needs to be examined.

### 4.1 Limitations

Although this study is the first to investigate the QET for people with mild to moderate intellectual disabilities or borderline intellectual functioning, there are some limitations that affect the interpretation of our results. First, our sample was relatively small, with the majority being Western‐European and having mild intellectual disabilities. This indicates that our findings need to be interpreted with care and seem to be less clear for other populations, that is, moderate intellectual disabilities and borderline intellectual functioning and/or with a different cultural background. We would recommend replication of this research in independent samples. Second, the time commitment for participants proved to be higher than expected, which often resulted in splitting up the assessment to keep it manageable for the participant and the interviewer. It may also have led to reduced willingness to participate and a higher dropout rate among participants, especially with regard to the second appointment to assess test–retest reliability. Third, low internal consistencies of the RFQ Other subscale and the AQ‐10 resulted in limited value for assessing the validity of the QET compared with these instruments. However, to the best of our knowledge, for mentalising and autistic traits, the adapted RFQ (Derks, Willemen, et al. 2023) and the adapted AQ‐10 (Kent et al. 2018) are the only questionnaires for people with intellectual disabilities studied on psychometric properties. Additionally, although correlations were as expected, the SED‐Q has not been validated yet for our population. This limits statements on the psychometric properties of the QET. Further research is encouraged to replicate the findings with improved instruments on reflective functioning on the self in relation to the feelings and thoughts of others and on autism/autistic traits (e.g., by employing the long questionnaire of the AQ [AQ‐50]) and a validated scale of emotional development completed together with people with intellectual disabilities. Fourth, the QET is a questionnaire assessing epistemic trust via self‐report. However, self‐report questionnaires are prone to overestimation (Finlay and Lyons 2001). Performance‐based measures can counterbalance the disadvantages of self‐report measures.

### 4.2 Further Research

Based on the findings of our study, the QET seems valuable to gain a preliminary understanding of important aspects of epistemic trust in people with mild to moderate intellectual disabilities or borderline intellectual functioning: Curiosity/openness, Expectation of help and Openness for help. As people with mild to moderate intellectual disabilities or borderline intellectual functioning often highly depend on care situations and relationships, it is important to have an instrument available that can assess these areas in order to improve the match between individual needs and provided care. Further development of the adapted QET for this population concerns, among other things, rephrasing of the items that did not load on any of the subscales. Specifically, it would be important to carefully test whether alternatives for difficult words and figurative speech mean to the reader what the author originally meant. The connotation of words related to vigilance can be positive and negative, and this comes very close when adapting the items. Additionally, studies into its psychometrics, including measurement invariance, in larger and more diverse samples with regard to intellectual functioning, cultural background and comorbid conditions (e.g., autism), would improve our understanding of the value in broader groups. To further strengthen construct validity, future research is needed to gain more insight into associations of the adapted QET with other measures on epistemic trust (the Epistemic Trust Assessment; Schröder‐Pfeifer et al. 2022; or the Epistemic Trust, Mistrust and Credulity Questionnaire; Campbell et al. 2021) and other measures assessing attachment (e.g., as described by Giltaij et al. 2017; Sterkenburg et al. 2021; using for example the Clinical Observation of Attachment behaviour; (Boris et al. 2004); Disturbances of attachment interview; Smyke and Zeanah 2022), mentalising (e.g., Certainty About Mental States Questionnaire; Müller et al. 2023) or social cognition (e.g., The Edinburgh Social Cognition Test; Baksh et al. 2018).

## 5 Conclusion

This study is the first to examine the possibility of using an adapted version of the QET as a self‐report measure for individuals with mild to moderate intellectual disabilities or borderline intellectual functioning. The psychometric properties indicate an adequate 4‐factor structure, though some items require further refinement. We found initial evidence for internal consistency and test–retest reliability. Correlations for construct validity suggest that the QET does not show substantial overlap with related constructs such as mentalising, emotional development and autism. Despite careful adaptations, our findings highlight the challenges of modifying figurative language to prevent literal misinterpretations within this population. Overall, this study provides valuable insights into improving the QET for future research and clinical applications.

## Author Contributions

P.S.S., A.B. and S.D.M.D. conceived and designed the project and were responsible for acquiring funding. S.D.M.D., S.K., V.M.M.A. and P.S.S. were responsible for adapting the questionnaire to fit the study context. S.D.M.D. and V.M.M.A. acquired the data. S.D.M.D., A.B. and V.M.M.A. analysed and interpreted the data. S.D.M.D. wrote the original draft; all authors contributed to writing and editing the paper. P.S.S. was responsible for the resources and project administration.

## Disclosure

This work does not contain any material reproduced from external sources.

## Ethics Statement

Ethical approval was granted by the Institutional Review Board of the Faculty of Behavioural and Movement Sciences of the Vrije Universiteit Amsterdam (VCWE‐2022‐140). The study was reviewed and approved under the provisions of the Medical Research Involving Human Subjects Act (WMO; 2022.0466).

## Consent

Before the study began, participants were required to read and sign a consent form. The form was read aloud to participants with visual impairments. In cases where participants were legally unable to give consent, their legal representative signed the agreement on their behalf. Supervisors, parents, or other involved caregivers were also asked to sign a consent form after completing a questionnaire about the participant. Additionally, a letter containing general information was given to the professional caregiver and legal representative to ensure they could help the participant make an informed decision. Participants were given a 2‐week period to consider their involvement. Independent researchers, including assistants and students, also signed a confidentiality agreement.

## Conflicts of Interest

The authors declare no conflicts of interest.

## Data Availability

The data that support the findings of this study are available on request from the corresponding author. The data are not publicly available due to privacy or ethical restrictions.
